# *Calanus finmarchicus* hydrolysate improves growth performance in feeding trial with European sea bass juveniles and increases skeletal muscle growth in cell studies

**DOI:** 10.1038/s41598-023-38970-5

**Published:** 2023-07-29

**Authors:** Isak Bøgwald, Tone-Kari K. Østbye, Alice Marie Pedersen, Sissel Beate Rønning, Jorge Dias, Karl-Erik Eilertsen, Sileshi Gizachew Wubshet

**Affiliations:** 1 The Norwegian College of Fishery Science, UIT—The Arctic University of Norway, P.O. box 6050, 9037 Tromsø, Norway; 2 Calanus AS, P.O. box 808, 9258 Tromsø, Norway; 3 Nofima AS—The Norwegian Institute of Food, Fisheries and Aquaculture Research, Osloveien 1, 1430 Ås, Norway; 4 SPAROS Lda, Área Empresarial de Marim, Lote C, 8700-221 Olhão, Portugal

**Keywords:** Cell growth, Animal physiology, Musculoskeletal models, Peptides

## Abstract

The world will be dependent on the development of novel feed ingredients from renewable sources to ensure sustainable growth of the aquaculture industry. Zooplankton like *Calanus finmarchicus* are viable new raw material candidates, as they have optimal nutrient profiles for aquatic animals and may be sustainably harvested in large volumes. In this study, the aim was to investigate if a protein hydrolysate of *C. finmarchicus* was able to influence the growth performance of fish. The effect of dietary inclusion of hydrolysates was tested in a feeding trial with European sea bass (*Dicentrarchus labrax*) juveniles, benchmarking calanus hydrolysate (CH) against commercially available hydrolysates. The diet with CH inclusion yielded increased growth, with significantly higher body weight than hydrolysates of sardine and tuna fish at the end of the trial. The observed growth-promoting effects were further examined using an in vitro model with skeletal muscle cells from Atlantic salmon. Through bioactivity experiments with muscle cells grown in media containing CH, low-molecular fractions were found to have the greatest positive effect on proliferation, viability, and expression of muscle-specific genes. Characterization of the most potent fraction revealed an abundance of small peptides, along with amino acids and marine metabolites associated with increased muscle growth.

## Introduction

The global population is projected to reach 9.8 billion by 2050^1^, which will require a corresponding increase in global food production. Terrestrial food sources are approaching their maximum sustainable capacity^2^, whereas only 7% of all proteins consumed globally come from seafood^3^. Seafood is rich in digestible protein and essential amino acids, and aquatic animals generally have a low feed conversion ratio (FCR) compared to terrestrial animals, which allows for high throughput of dietary protein^4^. The potential to feed the growing population with a larger share of seafood is obvious, especially as the demand for dietary protein sources with low environmental impact is expected to increase considerably in the coming years^5^.

In consideration of the uncertainty and present exploitation of many global fisheries, aquaculture is predicted to be the major source of seafood in the future^3,5^. Along with the expansion of aquaculture production comes questions about its sustainability, particularly with respect to the necessary feed ingredients. Fish meal has historically been the protein source of choice in aquaculture, but its seasonal variations and volume limitations have created needs for additional protein sources in the growing industry. The trend in recent years has thus been to formulate feeds with increasing amounts of terrestrial plant proteins such as soy, maize, and rapeseed^6^. Terrestrial plant protein sources have sub-optimal amino acid profiles compared to marine proteins, may contain anti-nutritional factors and mycotoxins, and generally display lower palatability^7–9^. These drawbacks are shown to reduce growth in farmed fish and have spurred demands for new and sustainable protein ingredients for aquaculture. Of the proposed novel ingredients, low-trophic marine resources such as zooplankton are viewed as attractive and viable options due to their renewable biomass volumes and their role as natural food in the marine food web. One zooplankton species with feed applications is *Calanus finmarchicus*, found across the Northern Hemisphere. In the Norwegian Sea and adjacent regions alone, the annual biomass production of *C. finmarchicus* and closely related species is estimated to be approximately 290 million tonnes, making it one of the most significant renewable resources in the region^10^. A recent review on mesopelagic species as new marine resources remarked that the biomass of *C. finmarchicus* is well understood and that the management plan, which issues ten commercial harvesting licenses for a total annual quota of 254,000 tonnes, governs its biological sustainability^11^. The massive volumes, combined with a suitable nutrient composition for aquatic animals^12,13^, make *C. finmarchicus* an especially promising raw material for novel aquaculture feed ingredients.

Enzymatic protein hydrolysis (EPH) is a method often used to process raw material into feed ingredients on an industrial scale. EPH is based on food-grade proteases to digest protein-rich biomasses, typically resulting in products that constitute a water-soluble peptide fraction, lipids, and a non-soluble residue solid fraction. This technology provides a gentle and environmentally friendly alternative to processing techniques with chemicals like solvents and acids. The protein fraction isolated after EPH is termed hydrolysate and can serve as an important ingredient in feed formulations for finfish^14^. In addition to their nutritional and functional properties, hydrolysates are potential sources of specific bioactive peptides that can have biological functions different from their precursor proteins. These bioactive peptides can display hormone or drug-like properties, and among the mode-of-actions they can exert are anabolic (growth-promoting), antioxidative, antimicrobial, antihypertensive, immunomodulatory, and opioid^15^. The inclusion of hydrolysates in diets for aquatic animals has been shown to promote growth and increase feed efficiency^16^, and a deeper look into specific fractions of the hydrolysates could help explain some of these bioactive effects. Previously published studies involving EPH of *C. finmarchicus* have focused on how to extract the oil phase most effectively^17^, there is thus little knowledge of the protein fraction and how it can benefit fish growth performance.

In the presented study, the overall aim was to investigate if a hydrolysate from *C. finmarchicus* could improve growth of the commercially important fish species European sea bass (*Dicentrarchus labrax*) and Atlantic salmon *(Salmo salar*). The first part consisted of a feeding trial that assessed how dietary inclusion of calanus hydrolysate influenced growth performance of European sea bass, benchmarking it against other commercial marine hydrolysates. The improved growth performance observed with calanus hydrolysate in the feeding trial was studied further in the second part, using a model of skeletal muscle cells from Atlantic salmon to identify bioactive fractions of the hydrolysate associated with increased skeletal muscle growth.

## Results and discussion

**Figure 1:**
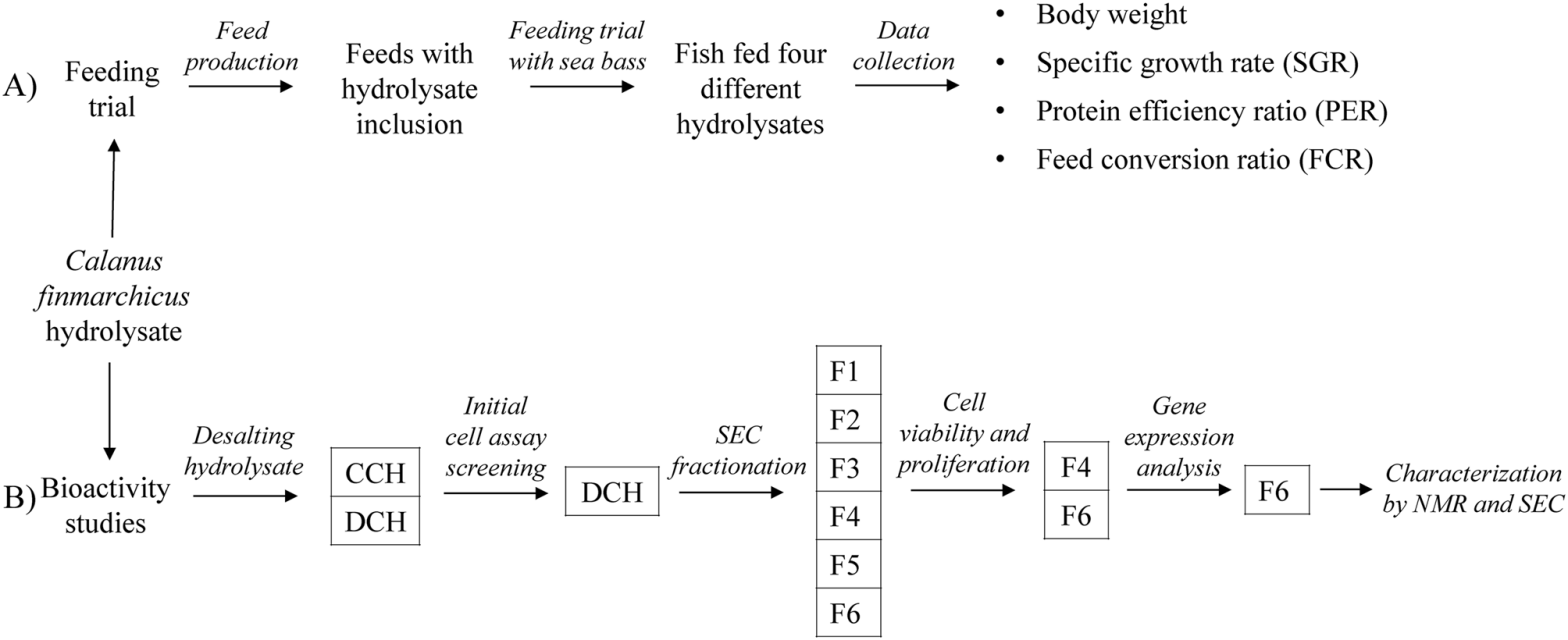
Schematic representation of the methods used to examine the growth-promoting properties of a hydrolysate from *Calanus finmarchicus*. (**A**) Feeding trial with European sea bass. (**B**) Bioactivity studies on calanus hydrolysate. CCH: Crude calanus hydrolysate; DCH: Desalted calanus hydrolysate; F1-F6: Fraction 1–6; SEC: Size exclusion chromatography; NMR: Nuclear magnetic resonance spectroscopy.

Dietary inclusion of a novel protein hydrolysate from the marine zooplankter *C. finmarchicus* led to improved growth performance of European sea bass in a feeding trial that benchmarked marine protein hydrolysates as functional feed ingredients. The observed growth-promoting properties of calanus hydrolysate (CH) were further evaluated using an in vitro cell model for skeletal muscle. Assessments of how supplementing CH in the cell culture medium affected viability, proliferation, and gene expression in cell studies, followed by fractionation of CH, led to the identification and characterization of the most bioactive fraction associated with increased muscle cell growth. A summary of the workflow is presented in Fig. 1.

### Feeding trial growth performance

The feeding trial aimed to benchmark the growth-promoting effects of dietary calanus hydrolysate (CH) against commercially available marine protein hydrolysates of sardine (SDH), tuna fish (TH), and salmon (SH). Moderate inclusion levels (5–10%) of marine protein hydrolysates to improve growth performance of fish have been well documented^14^. Marine hydrolysates may also enhance the nutritional properties to stimulate feed consumption^18^, and increase intake of nutrients and enhance growth, as observed in starter diets for Atlantic salmon^19^. Benchmarking CH against similar functional ingredients, thus employing diets with commercial marine hydrolysates as controls, would arguably yield more relevant results than comparing it to a standard fish meal control diet.

**Figure 2:**
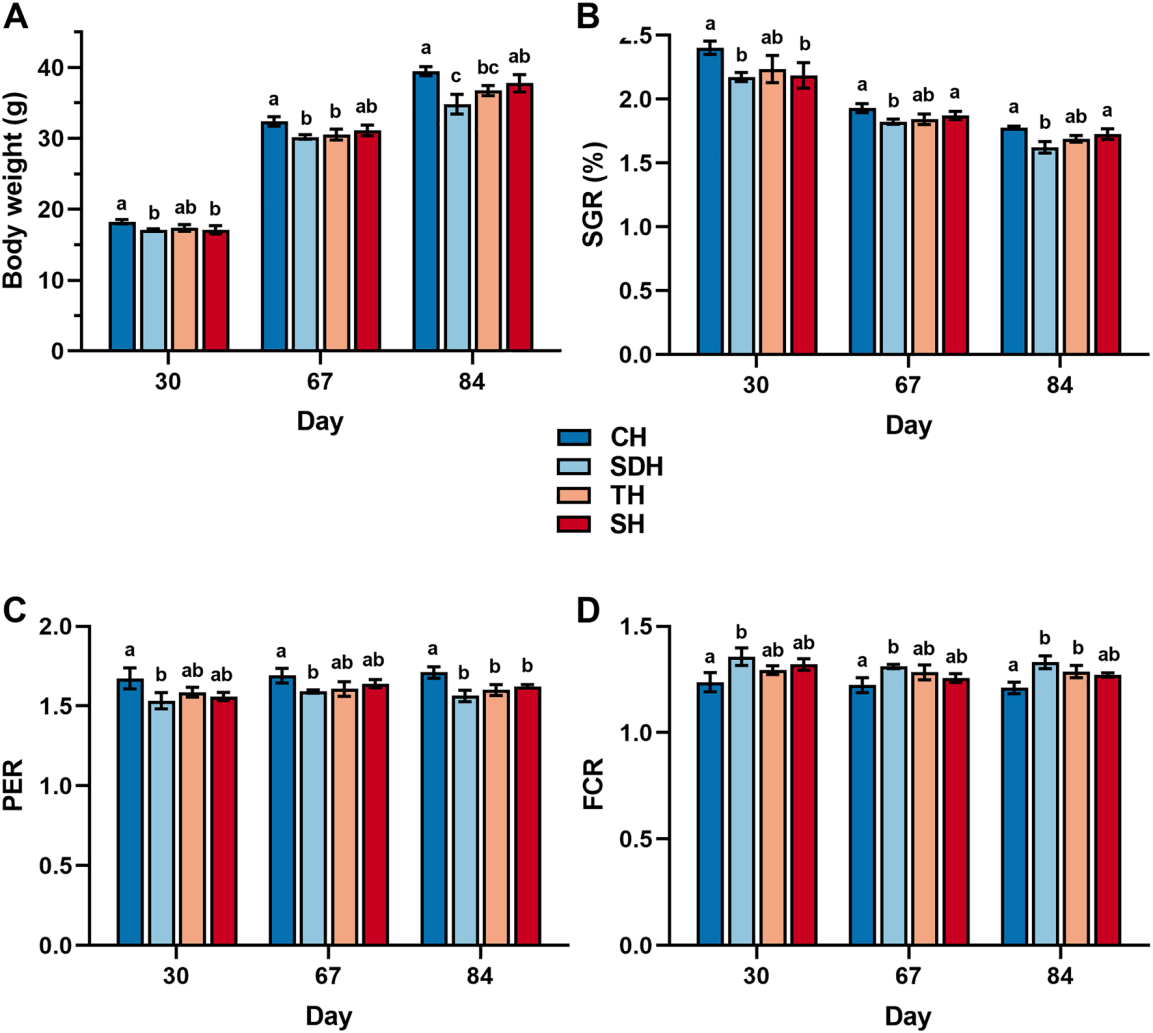
Benchmarking marine hydrolysates as feed ingredients for growth performance and feed utilization in European sea bass (*Dicentrarchus labrax*). (**A**) Body weight: fish were group weighed for each tank. (**B**) Specific growth rate (SGR): [(ln final weight – ln initial weight)/number of days] × 100. (**C**) Protein efficiency ratio (PER): wet weight gain/crude protein intake. (**D**) Feed conversion ratio (FCR): crude feed intake/weight gain. CH: Calanus hydrolysate; SDH: Sardine hydrolysate; TH: Tuna hydrolysate; SH: Salmon hydrolysate. Bars are means ± standard deviation (n = 3). Different letters denote statistical difference (*P* < 0.05).

European sea bass juveniles with a starting weight of 8.9 ± 0.4 g fully accepted all the diets. With similar starting weight for all the groups receiving the different diets and equal variances (Table S7.1–S7.3. in supplementary data), one-way ANOVA was applicable to compare the parameters at different time points. No significant effects were observed on survival, whole-body composition, or feed intake (Tables S3–S6 in supplementary data). However, CH inclusion yielded significantly higher body weight than SDH (*p* = 0.003) and TH (*p* = 0.047) after 84 days (Fig. 2A) and showed an increased growth rate compared with SDH (*p* = 0.002) throughout the trial (Fig. 2B). Growth performance is tightly connected with the ability to convert nutrients into body weight, and the relationship between increased body weight and consumed protein is the protein efficiency ratio (PER), which can be used to evaluate the quality of dietary protein ingredients. The fish receiving dietary CH showed higher PER throughout the trial (Fig. 2C), with significantly higher end results compared with the commercial hydrolysates SDH (*p* = 0.002), TH (*p* = 0.011), and SH (*p* = 0.037). Feed conversion ratio (FCR) measures the efficiency in which fish convert their input (feed) into the desired output (body weight), thus it takes the whole feed into account, not only the protein. At the end of the trial, feeds containing the three commercial hydrolysates had FCRs ranging from 1.28 to 1.33, whereas CH had an FCR of 1.22, significantly lower than those of SDH and TH (Fig. 2D). These FCR values correspond well with those found in similar studies with dietary hydrolysates for European sea bass^20^.

The results from the growth performance trial highlight the potential of CH as a functional ingredient in fish feeds, showing that dietary CH inclusion can improve growth and increase overall protein retention and feed conversion.

To the best of our knowledge, this is the first published study using a protein hydrolysate from *C. finmarchicus* in a feeding trial, there are thus few direct comparisons to be made. However, a study indicated that supplementation of *C. finmarchicus* meal in diets for juvenile Atlantic halibut (*Hippoglossus hippoglossus*) could greatly improve growth rate and nutrient utilization efficiency^21^. The meal was processed to increase its protein contents, but unlike optimal protein hydrolysates it also contained considerable amounts of lipids. Another study with zooplankton protein showed that dietary inclusion of Antarctic krill (*Euphausia superba*) meal for European sea bass enhanced growth performance and feed efficiency^22^. The evident benefits of including zooplankton such as *C. finmarchicus* and Antarctic krill in diets for fish should come as no surprise, as zooplankton are natural food for most fish species, especially in early life stages. However, little is known about why zooplankton and their products substantially benefit growth, and the subsequent experiments investigate these properties in further detail.

Considering that the diets were isonitrogenous, isolipidic, and isoenergetic (Table 3, Materials and methods), and that there were no significant differences in feed intake between them, any observed differences in growth performance would be based entirely on the composition and properties of the individual hydrolysates. Composition and properties of marine protein hydrolysates can be different depending on the raw material source, the enzymes used for hydrolysis, and the conditions and duration of hydrolysis^18^. Evidence that certain hydrolysate fractions can influence growth performance of fish in multiple ways have been found in several studies^23,24^.

### Effect of calanus hydrolysate on primary skeletal muscle cells

The first in vitro bioactivity experiment was performed to determine if the natural mineral contents of CH affected the growth of skeletal muscle, using a model with primary skeletal muscle cells from Atlantic salmon. European sea bass and Atlantic salmon are both teleost fish species and share the fundamental events of the myogenesis^25^. The salmon primary skeletal muscle cells are well-established models for bioactivity experiments to study muscle growth and its mechanisms^26–30^.

**Figure 3:**
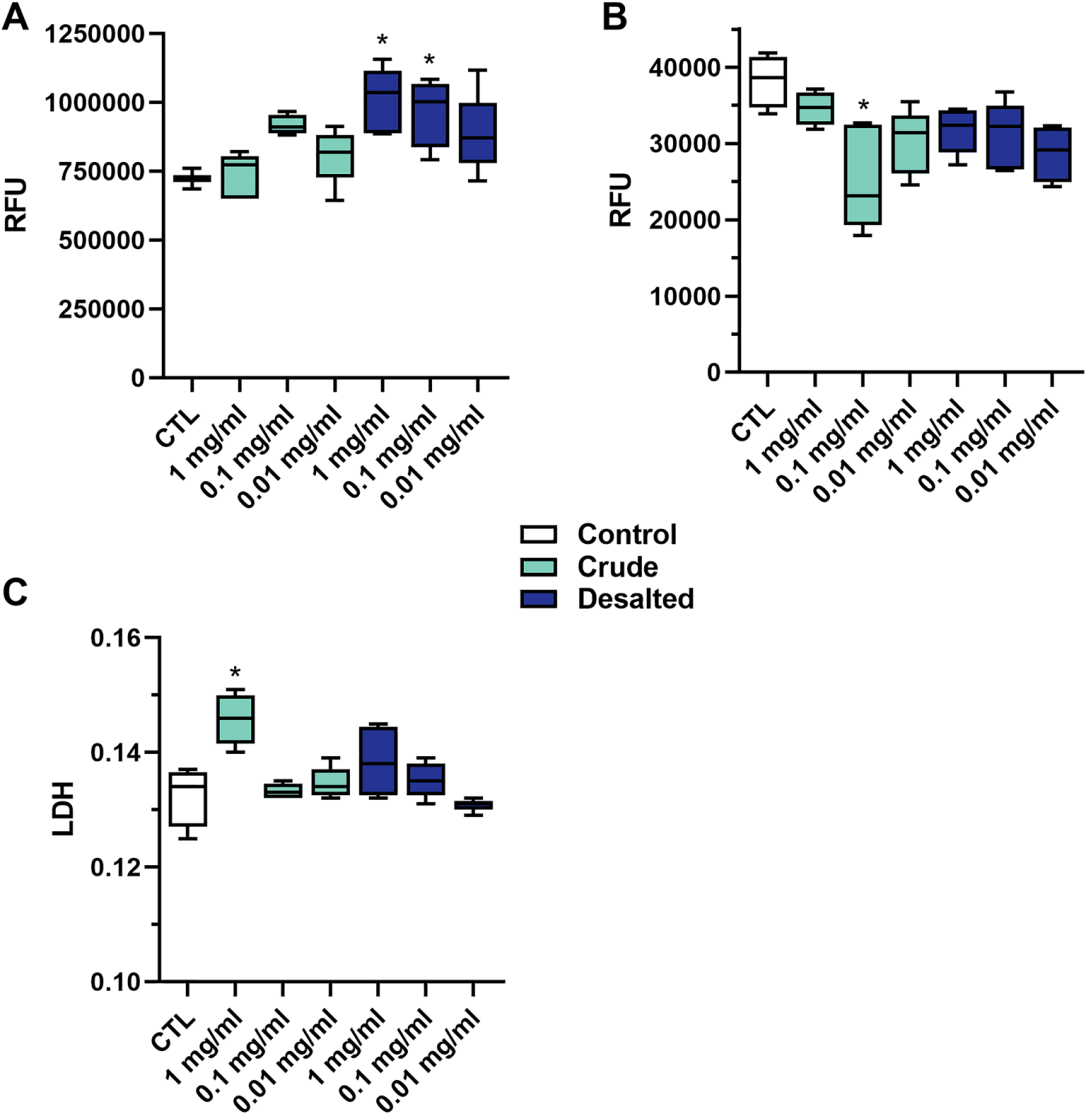
Viability (**A**), proliferation (**B**), and cytotoxicity (**C**) of Atlantic salmon muscle cells cultured in growth media supplemented with three concentrations (0.01, 0.1, or 1 mg/ml CH in growth medium) of crude or desalted calanus hydrolysate. CH: Calanus hydrolysate; RFU: Relative fluorescence units; LDH: lactate dehydrogenase; CTL: Control (no added hydrolysate to the cell culture growth media). Results presented with box (25^th^ to 75^th^ percentiles with line at median) and whiskers (min to max) plots (n = 4). Stars (*) denote statistically significant differences from control (*P* < 0.05).

Three concentrations of crude and desalted CH were added to growth media to test if supplementation of these could affect the muscle cell viability, proliferation, and cytotoxicity. The cell metabolic activity increased significantly when the cell culture media was supplemented with desalted CH at concentrations 1 mg/ml and 0.1 mg/ml (Fig. 3A), compared with cells treated with control medium (without added hydrolysates). The cell proliferation was not affected, except when cells were treated with culture medium containing crude CH at 0.1 mg/ml (Fig. 3B). Only the highest concentration of crude CH (1 mg/ml) supplemented in cell culture media induced increased lactate dehydrogenase (LDH) levels in the cells, which could impact cell survival negatively (Fig. 3C).

The initial bioactivity experiment showed that the desalted hydrolysates performed better in cell studies (Fig. 3). A putative explanation is that cultured cells are vulnerable to mineral contents higher than their innate concentrations, as high mineral concentrations can cause an osmotic effect where the cells lose water and shrink^31^. Whereas cultured cells can react negatively to elevated mineral contents, the opposite is true for most aquatic animals, especially those of marine origin, which require significant amounts of micronutrients like minerals in their diets^32^. Nonetheless, the results suggest the positive effects CH has on muscle growth stem from its inherent peptides, not from its mineral contents, and the desalted CH was thus chosen for the remaining in vitro experiments.

**Figure 4:**
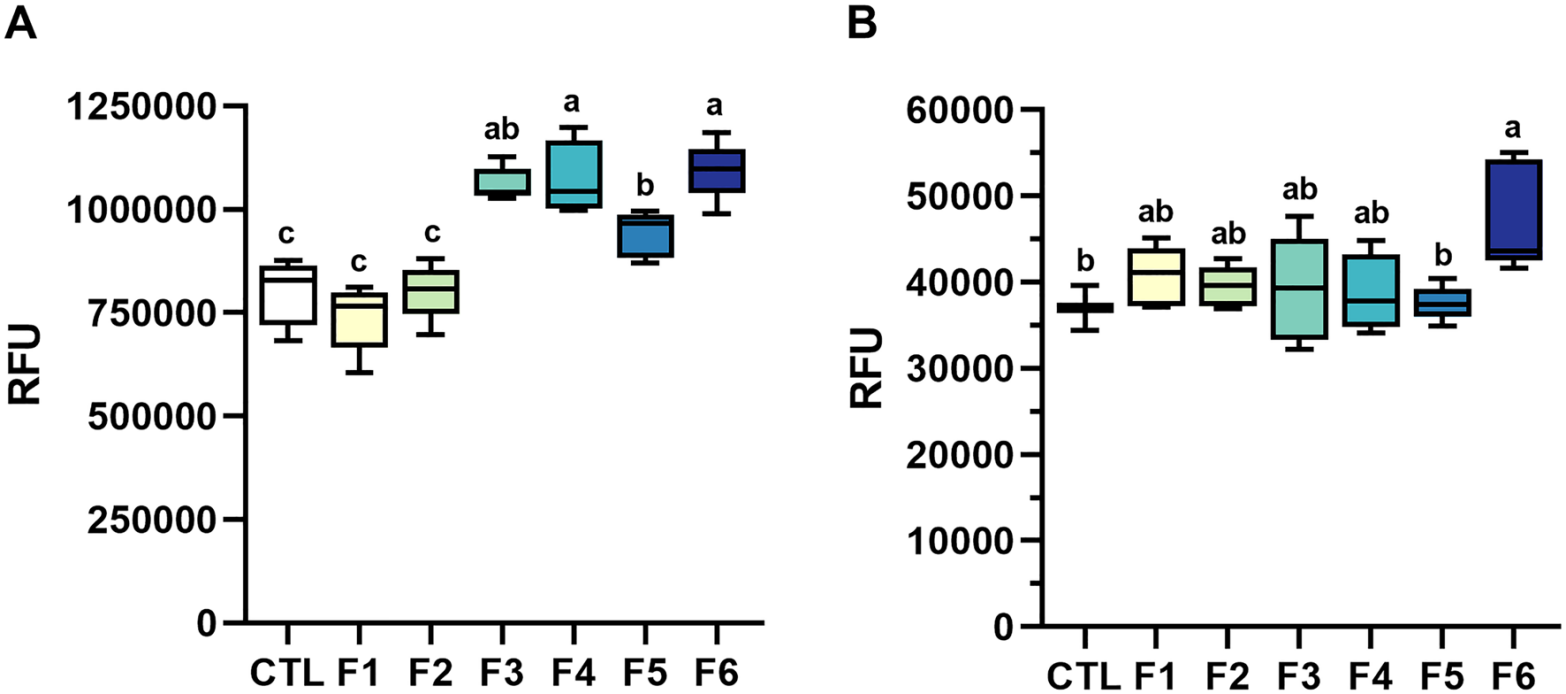
Viability (**A**) and proliferative activity (**B**) of Atlantic salmon muscle cells cultured in growth media supplemented with six size-separated fractions of desalted CH (0.1 mg/mL). RFU: Relative fluorescence units; CTL: Control; F1-F6: CH fractions 1–6. Results presented with box (25th to 75th percentiles with line at median) and whiskers (min to max) plots (n = 5). Lower case letters (a, b, c) denote statistically significant differences (*P* < 0.05).

As the next step to further narrow down the list of potentially bioactive compounds in CH, the desalted CH was fractionated to investigate how differently sized peptide fractions affect muscle growth. CH was separated using size-exclusion chromatography (SEC) and yielded six fractions with average molecular weights of  >742 Da (F1), 742 Da (F2), 527 Da (F3), 407 Da (F4), 316 Da (F5), and 260 Da (F6). The muscle cells were then cultured in growth media containing each of the peptide fractions to measure their individual influence, while the control cells were cultured in growth medium without supplementation of peptide fractions. F3, F4, and F6 were associated with the highest cell viability, significantly higher than the control and fractions F1 and F2, while F5 showed an intermediate effect (Fig. 4A). Likewise, cell culture medium supplemented with F6 induced the highest proliferative capacity, significantly higher than control and F5 (Fig. 4B). Overall, the results showed that fractions of lower molecular weight affected muscle growth more positively than those of higher molecular weight fractions. This is consistent with previous studies that showed growth-promoting effects of small molecular weight peptides^33^. Bakke et al.^23^ revealed that small peptides and amino acids formed during hydrolysis can facilitate the absorption of molecules in the intestinal tract by increasing the expression of peptide transporters. Rashidia, et al.^34^ found that low-molecular fractions from shrimp waste hydrolysate can improve growth performance of rainbow trout (*Oncorhynchus mykiss*), and Zheng et al.^24^ indicated a positive effect on growth and feed utilization in juvenile turbot (*Scophthalmus maximus*) by the use of low-molecular weight compounds from fish protein hydrolysate. Low-molecular weight fractions from protein hydrolysates have also been shown to be more bioactive in mammalian primary muscle cell studies^35,36^.

### Gene expression analysis

**Figure 5:**
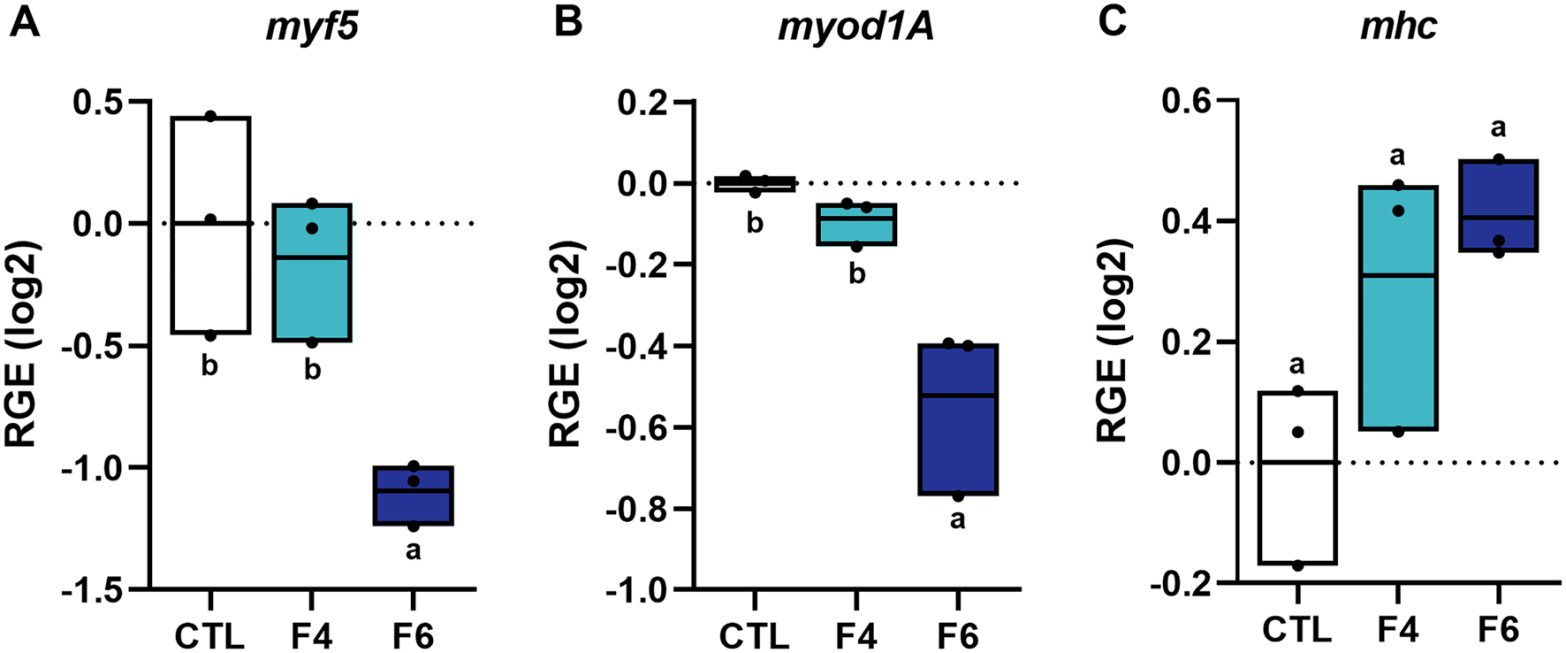
Relative gene expression of the muscle regulatory factors *myf5* (**A**) and *myod1a* (**B**) and the muscle structural protein *mhc* (**C**) upon 72 h of incubation with cell culture media supplemented with different CH fractions (F4 and F6). *myf5*: Myogenic factor 5; *myod1A*: Myoblast determination protein 1A; *mhc*: Myosin heavy chain; Relative gene expression (RGE); CTL: control, F4; CH fraction 4; F6: CH fraction F6. Results presented with box plots (min to max with line at mean, n = 3). Different letters denote statistical differences (*P* < 0.05).

The two most promising fractions from the viability (F4 and F6) and proliferation (F6) assays were chosen for gene expression analysis. Expression of genes associated with muscle growth was analyzed for cells cultured in media supplemented with F4 and F6, to explore the influence of these potent fractions in more detail. Proliferation and differentiation of myoblasts to multinuclear myotubes require the expression of genes coding for muscle regulatory factors such as myogenic factor 5 (Myf5) and myoblast determination protein 1a (MyoD1a)^37,38^. Myf5 and MyoD1a are early markers responsible for specification of cells to the muscle lineage^25^. The muscle regulatory factors are essential for the transcription of several muscle-specific genes, resulting in increased expression of the structural muscle proteins like myosin heavy chain (MHC). In differentiating muscle cells, early markers will be downregulated while late markers like structural muscle proteins will be upregulated^25^. Muscle cells cultured in growth media with F4 did not affect the gene expression of either *myf5* or *myod1a*, whereas F6 induced downregulation of both genes (Fig. 5A and B). Although the results were just below the statistical limit set for significance, there was a trend toward higher expression of *mhc* (Fig. 5C), with F6 showing the highest upregulation (*p* = 0.0518). A downregulation of the early muscle markers *myf5* and *myod1a*, concomitantly with a trending upregulation of *mhc*, may indicate that CH peptide fractions positively affected the muscle cells by increasing their rate of differentiation. The positive effects observed on in vitro muscle cell differentiation could be explained by the composition of certain small peptides and amino acids in F4 and F6. Supplementation of amino acids glutamate and arginine to in vitro cultured Atlantic salmon muscle cells has previously been found to influence gene expression of both early and late muscle-specific markers, including myogenin, Myf5, MyoD1a, Myf6 and myosin light chain^27^.

### Characterization of bioactive fraction

**Figure 6:**
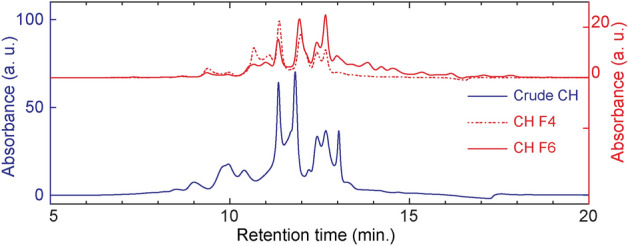
Molecular weight distribution of crude CH and fractions F4 and F6 acquired using size-exclusion chromatography with UV monitoring (214 nm).

The two low-molecular weight fractions of CH that showed the highest effect on viability (F4 and F6) and proliferation (F6) of Atlantic salmon muscle cells were further analyzed to study the molecular weight distribution of the peptides. Compared to crude CH, both F4 and F6 were shown to constitute relatively low amounts of early-eluting large proteins and high amounts of late-eluting smaller molecular weight peptides (Fig. 6). Average molecular weights of peptides in the fractions were calculated to be 407 and 260 g/mol for F4 and F6, respectively, which approximately correspond to peptides with an average length of 3.6 and 2.3 amino acid residues (calculated using a weighted average molecular weight of 113 g/mol for single amino acids). These results indicate that the growth-promoting effects observed in the feeding trial and in vitro studies can potentially be attributed to low-molecular weight peptides in CH, which are known to have efficient uptake by peptide transporters in the intestines^39^ and improve growth performance^34^.

**Figure 7:**
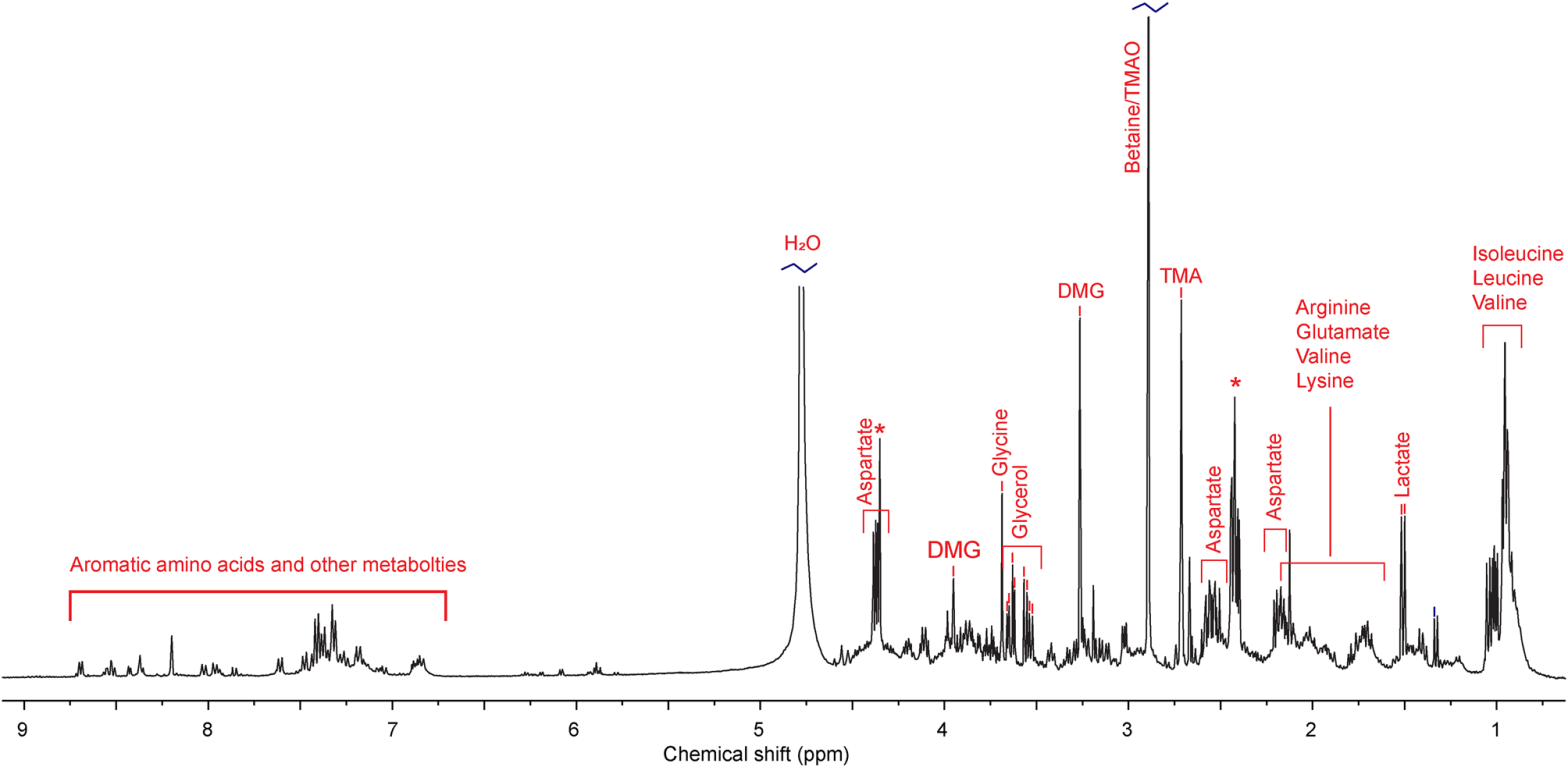
^1^H NMR spectra of CH-F6 with tentative assignments based on previously reported chemical shift values. Assignments were done for the abundant peaks, and only peaks diagnostic to a given molecule were assigned. Unassigned peaks are marked with “*”.

Identified as the most bioactive fraction in terms of viability, proliferation, and gene expression of in vitro cultured muscle cells, F6 was further characterized with ^1^H NMR spectroscopy (Fig. 7). Tentative assignments of the proton NMR peaks were performed according to published chemical shift values for closely related samples^40–42^. In addition to characteristic signals assigned to side chains of aromatic and aliphatic amino acids, significantly large signals were assigned to common marine metabolites such as trimethylamine *N*-oxide (TMAO) and dimethyl glycine (DMG). The abundance of DMG in combination with the branched-chain amino acids (BCAAs) was particularly interesting. Supplementation of DMG-salt in diets has been shown to improve growth performance and skeletal muscle function in terrestrial animals^43,44^ and inclusion of dietary BCAAs in aquafeeds to contribute to growth and flesh quality^45^. Glutamate and arginine, the aforementioned amino acids found to increase the expression of muscle-specific genes^27^, were also identified as part of several abundant peaks in the fraction. Lactate is yet another metabolite identified in the fraction that has been associated with growth stimulation. It has been reported to play a role in the exercise-induced human growth hormone response^46^, to stimulate hypertrophy and regeneration of mouse skeletal muscle^47^, and to stimulate growth when supplemented to the diet of Arctic charr (*Salvelinus alpinus*)^48^.

## Conclusions

Dietary inclusion of calanus hydrolysate (CH) in a feeding trial with European sea bass juveniles led to increased growth performance and feed efficiency compared with other commercial marine hydrolysates, highlighting the potential of CH as a functional ingredient in fish feed. In the following in vitro study, low-molecular weight fractions of CH were associated with increased viability and proliferative activity in a salmon skeletal muscle cell model. Furthermore, a gene expression experiment showed downregulation of early muscle markers and a trend towards higher expression of a structural muscle marker, which may indicate a positive effect on muscle differentiation. Characterization of the most bioactive fraction by ^1^H NMR revealed that it contained small peptides and important amino acids, but also metabolites previously found to be associated with increased muscle growth. Further studies are required to quantify the direct contribution of such metabolites to the observed growth-promoting effects. To the authors knowledge, the present study is the first to demonstrate the promising potential of CH as a feed ingredient with specific growth-promoting constituents.

## Materials and methods

### Marine hydrolysate test products

*Calanus finmarchicus* raw material was harvested and processed into calanus hydrolysate (CH) by Calanus AS (Tromsø, Norway), and the commercial hydrolysates from salmon (SH), sardine (SDH), and tuna fish (TH) were bought and supplied by SPAROS, Lda. (Olhão, Portugal). Calanus and salmon hydrolysates were used in liquid form, while those of sardine and tuna were dry powders. The nutrient composition of the test hydrolysates is presented in Table 1.

**Table 1:** Nutrient composition of test hydrolysates.

Hydrolysate	CH	SDH	TH	SH
Product form	Liquid	Powder	Powder	Liquid
Moisture, %	46.7	3.3	3.2	51.2
Crude protein, %	63.0	82.2	78.8	71.7
Crude fat, %	1.1	0.2	7.4	8.0
Ash, %	20.5	16.1	13.5	10.5
Phosphorus, %	1.48	0.97	0.84	2.50
Gross energy, kJ/g	18.4	19.0	22.2	21.9
Arginine, %	4.18	3.50	4.75	4.34
Histidine, %	0.86	1.94	2.20	1.78
Isoleucine, %	2.27	1.45	2.27	2.91
Leucine, %	3.88	3.22	4.33	4.69
Lysine, %	4.24	3.69	5.53	5.00
Threonine, %	2.31	1.79	3.12	2.91
Valine, %	2.96	2.10	3.19	3.55
Methionine, %	1.16	0.73	1.49	1.97
Cysteine, %	0.62	0.31	0.57	0.76
Phenylalanine, %	2.03	1.55	2.91	2.30
Tyrosine, %	2.23	0.63	0.85	2.11
Aspartic acid, %	4.80	4.26	6.24	5.78
Glutamic acid, %	7.22	7.65	9.79	8.01
Alanine, %	3.98	5.67	6.38	4.30
Glycine, %	4.30	10.03	10.57	5.29
Proline, %	2.12	4.11	5.75	3.52
Serine, %	2.08	1.94	2.98	2.79
Taurine, %	1.05	0.53	0.30	0.53

All values, except moisture, are given as percentage of dry matter. CH: Calanus hydrolysate; SDH: Sardine hydrolysate; TH: Tuna fish hydrolysate; SH: Salmon hydrolysate.

### Formulation of experimental diets

The four experimental diets in the trial were formulated with low levels of fish meal and wheat gluten, compensated with the marine hydrolysate test products (calanus, salmon, sardine, and tuna fish) and additional plant proteins (Table 2). To make all the diets equivalent in terms of protein supply, the inclusion level of the hydrolysates ranged from 2.95% to 4.20% (dry basis) based on their different crude protein content (Table 1). Overall, the formulated diets were isonitrogenous (48% crude protein), isolipidic (16% crude fat) and isoenergetic (21.9 MJ/kg).

**Table 2:** Formulation of the experimental diets.

Ingredients, %	CH	SDH	TH	SH
Fishmeal Super Prime^a^	5.00	5.00	5.00	5.00
Fishmeal 60^b^	5.00	5.00	5.00	5.00
Calanus hydrolysate (dry basis)^c^	4.20			
Salmon hydrolysate (dry basis)^c^				3.55
Sardine hydrolysate^c^		2.95		
Tuna hydrolysate^c^			3.25	
Brewer's yeast^d^	2.50	2.50	2.50	2.50
Soy protein concentrate^e^	15.00	15.00	15.00	15.00
Wheat gluten^f^	8.50	8.50	8.50	8.50
Corn gluten meal^g^	15.00	15.00	15.00	15.00
Soybean meal 48^h^	16.00	16.00	16.00	16.00
Rapeseed meal^i^	7.50	7.50	7.50	7.50
Wheat meal^j^	0.80	2.35	2.15	1.95
Whole peas^k^	3.50	3.50	3.50	3.50
Vitamin & Mineral premix^l^	1.00	1.00	1.00	1.00
Vitamin E50^m^	0.05	0.05	0.05	0.05
Betaine HCl^n^	0.15	0.15	0.15	0.15
Antioxidant^o^	0.20	0.20	0.20	0.20
Monosodium phosphate^p^	1.80	1.80	1.70	1.60
DL-Methionine^q^	0.20	0.20	0.20	0.20
Soy lecithin^r^	1.00	1.00	1.00	1.00
Fish oil^s^	6.50	6.50	6.50	6.50
Rapeseed oil^t^	6.10	5.80	5.80	5.80

^a^Super Prime: 66.3% CP, 11.5% CF, Pesquera Diamante, Peru.

^b^COFACO 60: 62.3% crude protein (CP), 8.4% crude fat (CF), COFACO, Portugal.

^c^Please check details in Table 1.

^d^Brewer’s yeast: 39% CP, Premix Lda, Portugal.

^e^Soycomil P: 62% CP, 0.7% CF, ADM, The Netherlands.

^f^VITEN: 82% CP, 2.1% CF, Roquette, France.

^g^Corn gluten meal: 61% CP, 6% CF, COPAM, Portugal.

^h^Dehulled solvent extracted soybean meal: 47% CP, 2.6% CF, CARGILL, Spain.

^i^Defatted rapeseed meal: 32.7% CP, 4.1% CF, Ribeiro & Sousa Lda, Portugal.

^j^Wheat meal: 11.7% CP, 1.6% CF, MOLISUR, Spain.

^k^Yellow peas: 19% CP, 2% CF, Ribeiro e Sousa Lda, Portugal.

^l^PREMIX Lda, Portugal. Vitamins (IU or mg/kg diet): DL-alpha tocopherol acetate, 100 mg; sodium menadione bisulphate, 25 mg; retinyl acetate, 20,000 IU; DL-cholecalciferol, 2000 IU; thiamine, 30 mg; riboflavin, 30 mg; pyridoxine, 20 mg; cyanocobalamin, 0.1 mg; nicotinic acid, 200 mg; folic acid, 15 mg; ascorbic acid, 1000 mg; inositol, 500 mg; biotin, 3 mg; calcium pantothenate, 100 mg; choline chloride, 1000 mg, betaine, 500 mg. Minerals (g or mg/kg diet): cobalt carbonate, 0.65 mg; copper sulphate, 9 mg; ferric sulphate, 6 mg; potassium iodide, 0.5 mg; manganese oxide, 9.6 mg; sodium selenite, 0.01 mg; zinc sulphate, 7.5 mg; sodium chloride, 400 mg; calcium carbonate, 1.86 g; excipient wheat middlings.

^m^ROVIMIX E50, DSM Nutritional Products, Switzerland.

^n^Beta-Key 95%, ORFFA, The Netherlands.

^o^VERDILOX PX, KEMIN EUROPE NV, Belgium.

^p^Bolifor MSP: 24% P, Yara International, Norway.

^q^Rhodimet NP99, ADISSEO, France.

^r^LECICO GmbH, Germany.

^s^Sopropêche, France.

^t^JC Coimbra, Portugal. CH: Calanus hydrolysate; SDH: Sardine hydrolysate; TH: Tuna fish hydrolysate; SH: Salmon hydrolysate.

### Manufactured diets

Diets were produced by extrusion at SPAROS. All powder ingredients were mixed according to the target formulation in a double-helix mixer (model RM90, MAINCA, Spain) and ground (below 250 µm) in a micropulverizer hammer mill (model SH1, Hosokawa-Alpine, Germany). Test hydrolysates from calanus and salmon, since presented in the liquid form, were incorporated in the feed mash after grinding. Diets (pellet sizes: 1.5 and 2.0 mm) were manufactured with a twin-screw extruder (model BC45, Clextral, France) with a screw diameter of 55.5 mm. Extrusion conditions were as follows: Feeder rate (44 kg/h), screw speed (142 rpm), water addition (approximately 290 ml/min), temperature barrel 1 (24–29 °C), temperature barrel 3 (105–107 °C). Extruded pellets were dried in a vibrating fluid bed dryer (model DR100, TGC Extrusion, France). After cooling, oils were added post-extrusion by vacuum coating (model PG-10VCLAB, Dinnissen, The Netherlands). Completed pellets were packed in sealed plastic buckets and shipped to the research site where they were stored at room temperature, in a cool and well-aerated environment. Samples of each diet were taken for analytical characterization (Table 3, Tables S1-S2 in supplementary data). Analysis of complete feeds were carried out with analytical duplicates, following the methodology described by AOAC^49^ for most analytics: Dry matter after drying at 105 °C for 24 h; total ash by combustion (550 °C, 6 h) in a muffle furnace (Nabertherm L9/11/B170, Germany); crude protein (N × 6.25) by a flash combustion technique followed by gas chromatographic separation and thermal conductivity detection with a Leco N analyser (Model FP-528, Leco Corporation, USA); crude lipid by petroleum ether extraction (40–60 °C) using a Soxtherm system (Multistat SX PC, Gerhardt, Germany); gross energy in an adiabatic bomb calorimeter (Werke C2000, IKA, Germany). Amino acids were determined after hydrolysis in 6 M HCL at 108 °C for 24 h in nitrogen-flushed vials, using a Waters Pico-Tag reversed-phase HPLC system with norleucine as an internal standard. The resulting chromatograms were analyzed with Breeze software (Waters, USA). Tryptophan was not analyzed due to its destruction by acid hydrolysis. Minerals and fatty acids were analyzed at a certified laboratory (Eurofins Anàlisis Alimentario SL, Spain), according to ISO 27,085:2009 by ICP-AES methodology^50^ for minerals, and by gas-chromatography and flame ionization detection (GC-FID) for fatty acids.

**Table 3:** Analytical composition of experimental diets (as fed basis).

	CH	SDH	TH	SH
Moisture, %	5.4 ± 0.0	5.5 ± 0.0	3.3 ± 0.0	3.9 ± 0.1
Ash, %	7.1 ± 0.4	6.9 ± 0.1	7.1 ± 0.0	6.9 ± 0.1
Crude protein, %	48.4 ± 0.1	48.1 ± 0.1	48.7 ± 0.0	48.5 ± 0.0
Crude fat, %	16.1 ± 0.1	15.8 ± 0.0	16.3 ± 0.1	16.5 ± 0.1
Gross energy, kJ/g	21.7 ± 0.0	21.8 ± 0.0	22.2 ± 0.0	22.1 ± 0.0
Arginine, %	2.42 ± 0.01	2.46 ± 0.02	2.38 ± 0.01	2.58 ± 0.02
Histidine, %	1.04 ± 0.01	0.99 ± 0.01	1.12 ± 0.01	1.13 ± 0.01
Isoleucine, %	1.82 ± 0.01	1.78 ± 0.01	1.85 ± 0.01	1.93 ± 0.01
Leucine, %	4.17 ± 0.02	4.11 ± 0.02	4.24 ± 0.03	4.30 ± 0.01
Lysine, %	2.13 ± 0.01	2.13 ± 0.02	2.29 ± 0.01	2.23 ± 0.01
Threonine, %	1.65 ± 0.01	1.67 ± 0.01	1.76 ± 0.03	1.70 ± 0.01
Tryptophan, %	0.48 ± 0.01	0.46 ± 0.01	0.49 ± 0.01	0.47 ± 0.00
Valine, %	2.16 ± 0.01	2.06 ± 0.01	2.24 ± 0.03	2.23 ± 0.01
Methionine, %	0.97 ± 0.06	1.03 ± 0.02	1.06 ± 0.01	1.08 ± 0.01
Cysteine, %	0.61 ± 0.01	0.60 ± 0.02	0.62 ± 0.01	0.62 ± 0.01
Phenylalanine, %	2.29 ± 0.01	2.31 ± 0.01	2.35 ± 0.01	2.38 ± 0.02
Tyrosine, %	1.69 ± 0.03	1.70 ± 0.02	1.69 ± 0.01	1.66 ± 0.01
Aspartic acid, %	3.60 ± 0.04	3.72 ± 0.02	3.99 ± 0.05	3.84 ± 0.03
Glutamic acid, %	9.83 ± 0.02	9.25 ± 0.05	9.70 ± 0.05	9.53 ± 0.05
Alanine, %	2.46 ± 0.01	2.47 ± 0.03	2.53 ± 0.02	2.54 ± 0.03
Glycine, %	2.08 ± 0.01	2.13 ± 0.02	2.16 ± 0.01	2.13 ± 0.01
Proline, %	3.19 ± 0.03	3.06 ± 0.02	3.33 ± 0.01	3.23 ± 0.01
Serine, %	2.18 ± 0.01	2.21 ± 0.01	2.28 ± 0.02	2.24 ± 0.01

Values are means ± standard deviation (n = 2). CH: Calanus hydrolysate; SDH: Sardine hydrolysate; TH: Tuna fish hydrolysate; SH: Salmon hydrolysate.

### Ethical statement

The feeding trial was conducted at the experimental facilities of SPAROS (Olhão, Portugal), which are registered for experimentation with aquatic species by Direção-Geral de Alimentação e Veterinária (Ministry of Agriculture, Portugal). The experimental protocol was approved by the Animal Welfare Committee—Órgão Responsável pelo Bem-Estar Animal (ORBEA) of SPAROS (Trial reference: CALBASS). Experiments were conducted by FELASA certified scientists and technical staff, in full compliance with ARRIVE guidelines and the European (Directive 2010/63/EU) and Portuguese (Decreto-Lei nº. 113/2013, August 7th) legislation on the protection of animals for scientific purposes.

### Feeding trial

The trial was performed at the experimental research facilities of SPAROS (Olhão, Portugal), with European sea bass (*Dicentrarchus labrax*) sourced at Estação Piloto de Piscicultura de Olhão (EPPO) from the Portuguese Institute of the Sea and Atmosphere (IPMA). Approximately 650 fish were transferred to the SPAROS experimental facilities by a duly authorized carrier and no significant mortality or pathological signs were observed in association with transport. The fish were kept in sanitary quarantine for three weeks, during which the fish were maintained and fed a standard commercial diet, before the start of the trial.

A total of 360 fish were distributed into 12 tanks (30 fish per tank), with three tanks for each of the experimental diets. Four experimental diets were formulated (Table 2), each with the inclusion of a marine hydrolysate (calanus, sardine, tuna, or salmon). The mean initial body weight of the fish was 8.9 ± 0.4 g, and they were fed the experimental diets for 84 days. The sub-square PVC tanks contained 90 L of water and were located indoors, supplied with flow-through saltwater (flow rate: 4.6 L/min), and subjected to photoperiod conditions of 12 h light and 12 h dark. The average water temperature during the trial was 23.0 ± 1.4 °C, salinity was 35.8 ± 0.5 ‰, dissolved oxygen levels in the water were kept above 5.3 mg/L, and ammonia levels were below the detection limit.

Fish were hand-fed to visual satiety in four daily meals at 09:00, 12:00, 15:00, and 18:00. Utmost care was taken to avoid feed wastage and allow precise quantification of intake. Lightly anesthetized (20 mL/L of AQUI-S, New Zealand) fish were group weighed at the start, day 30, day 67, and finally at day 84. Feed intake was calculated, survival was monitored daily, and growth performance was recorded on the abovementioned days. Feed conversion ratio (FCR), protein efficiency ratio (PER), and specific growth rate (SGR) were calculated (*n* = 3) upon completion of the trial using these formulas:

- FCR: crude feed intake/weight gain.
- PER: wet weight gain/crude protein intake.
- SGR: (Ln final body weight – Ln initial body weight) × 100/days.

### Clean-up and fractionation of calanus hydrolysates

Crude calanus hydrolysate (CCH, 46% dry matter) was freeze-dried to afford a light brown powder. To further refine peptides from the crude hydrolysate, CCH was diluted 1:1 in Milli-Q water, filtered using Millex syringe filter with a PVDF membrane (pore size 0.45 μm, Merk Millipore, Burlington, MA, USA), and desalted using semi-preparative chromatography. Fractionations were performed using a Dionex Ultimate 3000 instrument (Thermo Scientific, Waltham, MA, USA) equipped with a quaternary pump and thermometer auto-sample, and a variable wavelength UV-detector. Desalting was performed using a reversed-phase Thermo Betasil C18 column, 250 × 10 mm i.d., 10 μm particle size (Thermo Scientific). The mobile phase used was H_2_O (Solvent A) and acetonitrile (Solvent B), both acidified with 0.1% formic acid. The following elution profile was used at a flow rate of 2 mL/min: 0 min, 0% B; 10.0 min, 0% B; 35.0 min, 100% B; 41.0 min, 100% B; 51.0 min, 0% B. The desalted calanus hydrolysate (DCH) fractions were collected between 5.2 min and 35.0 min retention time and freeze-dried to afford light yellow powder. Both freeze-dried CCH and DCH were directly used for the initial screening in the cell culture assays. DCH was further fractionated using size exclusion chromatography (SEC). Fractionation based on molecular weight was performed using the same system described above and a preparative Yarra SEC-2000 column, 300 × 21.1 mm i.d., 5 µm particle size (Phenomenex, Torrence, CA, USA). The mobile phase consisted of a mixture of acetonitrile and ultrapure water in a proportion of 30:70 (v/v), containing 0.1% formic acid. CCH diluted 1:1 in Milli-Q water filtered using Millex syringe filter with a PVDF membrane was used as an injection solution. The chromatographic separation was performed using isocratic elution with 30% acetonitrile (0.1% formic acid) at a flow rate of 3 mL/min from 0 to 55 min. This was followed by washing for 3 min with NaH_2_PO_4_ (0.10 M) and subsequent column equilibration of 20 min with 30% acetonitrile. Fractions were collected from a total of 15 injections of 500 μL. A total of six fractions CH-F1 to CH-F6 were collected, pooled, and freeze-dried prior to analysis with cell cultures. The active fractions (F4 and F6) identified from the cell culture study were analysed for molecular weight distribution using the protocol by Wubshet et al.^51^.

### Isolation and culturing of primary Atlantic salmon skeletal muscle cells

Atlantic salmon primary skeletal muscle cells were isolated according to Østbye and Ytteborg ^28^. The isolated muscle cells were seeded in T25 cell flasks (muscle tissue from eight 5 cm salmon per T25 cell flask) with growth media containing 10% fetal bovine serum (FBS), 0.01 M HEPES (Sigma-Aldrich, St. Louis, USA), 10 mL/L antibiotic–antimycotic (Sigma-Aldrich, St. Louis, USA), in L-15 medium GlutaMAX (Invitrogen, Carlsbad, USA). The cells were incubated at 13 °C without CO_2_ for 2 days. The cells were then trypsinated and transferred to 96-well plates for viability or proliferation assays or to 6-well plates for gene expression analysis. After one day incubation at 13 °C without CO_2_, the cells were added growth medium containing 2% FBS and the different concentrations of calanus hydrolysate. The cells were incubated with hydrolysates for 72 h before harvesting to the different analyses.

Two separate cell trials were conducted to investigate the effect of calanus hydrolysate on Atlantic salmon skeletal muscle growth. In the first cell trial, three concentrations (1.00, 0.10 and 0.01 mg/mL, pH7) of crude and desalted calanus hydrolysates were tested on viability, toxicity, and proliferation of cells. In the second cell trial, six different size separated fractions (F1-F6) of the desalted hydrolysate (0.10 mg/mL) were investigated by viability and proliferation assays. Only F4 and F6 were included for the gene expression analysis.

### Cell viability, toxicity, and proliferation

Viability of the cells was evaluated by measuring ATP production with CelltiterGlo (Promega Corporation, Madison, WI, USA), potential toxicity of the hydrolysates using Cytotoxicity detection kit (LDH) (Roche Diagnostics GmbH, Mannheim, Germany), and proliferation of the cells using CyQUANT Cell Proliferation Assay (Molecular Probes, Oregon, USA), following the manufacturer’s protocols.

### Gene expression analysis

RNeasy kit (Qiagen, Valencia, CA, USA) was used to isolate total RNA from the muscle cells following the producer’s protocol. All samples were treated with DNase I (Invitrogen, Carlsbad, USA) to remove genomic DNA. The concentration and purity of the RNA were evaluated using NanoDrop 1000 Spectrophotometer (NanoDrop Technologies, USA). Taqman reverse transcriptase reagents (Applied Biosystems, Foster City, CA, USA) were used to synthesize cDNA from 500 ng RNA in a 20 μL reaction volume according to the following conditions: 25 °C for 10 min, 37 °C for 30 min, and 95 °C for 5 min. The qPCR reaction mixture consisted of 4 μl diluted (1:10) cDNA, 1 μl forward and reverse primer at a final concentration of 0.5 μM (Table S8 in supplementary data), and 5 μl PowerUp SYBR Green Master Mix (Applied Biosystems, Foster City, California, United States). A standard curve was included for each primer pair to evaluate the primer efficiency, and a melting curve analysis was included to verify the amplification of only one product. All samples were analyzed in parallel, and non-template and non-enzyme controls were included. The qPCR reaction was run on a QuantStudio 5 instrument (Thermo Fisher Scientific, MA, USA) under the following conditions: 50 °C for 20 s, 95 °C for 20 s, 40 cycles with 95 °C for 1 s and 60 °C for 20 s, 95 °C for 1 s and 60 °C for 20 s, 95 °C for 1 s. RefFinder^52^ was used to evaluate the most stable reference gene of *ef1a*, *rpol2* and *etif3*. The relative gene expression level was calculated according to the ΔΔCt method with efficiency correction^53^ using *rpol2* as a reference gene.

### NMR experiment

^1^H NMR analysis of the promising fraction (F6) was performed using a Bruker Ascend system (^1^H operating frequency of 400 MHz) equipped with a sample changer and a gradient inverse triple-resonance 5 mm probe-head (Bruker Biospin). NMR sample was prepared by dissolving appx. 5 mg of CH-F6 in 0.7 mL D_2_O containing 0.05 wt. % TMSP-*d*4 sodium salt as an internal standard. IconNMR (version 4.2, Bruker Biospin) was used for controlling automated acquisition of NMR data (temperature equilibration to 300 K, optimization of lock parameters, gradient shimming, and setting of receiver gain), and processing of NMR data was performed using Topspin (version 3.0, Bruker Biospin). The one-dimensional ^1^H NMR spectra were acquired using 30° pulses, 4 s acquisition time, 1 s relaxation delay and 32 K data points. The data points were zero-filled to 64 K and multiplied with an exponential function (line-broadening = 0.3 Hz) prior to Fourier transformation.

### Statistical analysis

Evaluation criteria data of the feeding trial are presented as means ± standard deviation, with n = 3 replicates. Data from the bioactivity studies with skeletal muscle cells are presented with box-plots (n = 3–5 replicates), to better represent the distribution of these specific data. All data were normally distributed with equal variances (Table S7.1–S7.3 in supplementary data). Starting weight of the fish in the different diet groups were similar, allowing a one-way analysis of variance (ANOVA) for comparison of growth parameters at different time points. One-way ANOVA was also performed for all the bioactivity studies with skeletal muscle cells. When appropriate, means were compared with the Tukey method. Statistical significance was tested at a 0.05 probability level. All statistical tests were performed using GraphPad Prism (version 9.2.0).

## Supplementary Information

Supplementary Information.

## Data Availability

The gene expression datasets generated and/or analyzed during the current study are available in the DataverseNO repository for open research data (https://doi.org/10.18710/MRVDNS), all other data can be found in the supplementary material or from the author upon request.

## Abbreviations

BCAA: Branched-chain amino acid
CH: Calanus hydrolysate
CCH: Crude calanus hydrolysate
CTL: Control
DCH: Desalted calanus hydrolysate
DMH: Dimethyl glycine
EPH: Enzymatic protein hydrolysis
F1–F6: Fraction 1–6 of calanus hydrolysate
FCR: Feed conversion ratio
MHC: Myosin heavy chain
Myf5: Myogenic factor 5
MyoD1a: Myoblast determination protein 1a
NMR: Nuclear resonance magnetic spectroscopy
PER: Protein efficiency ratio
RFU: Relative fluorescence units
SH: Salmon hydrolysate
SDH: Sardine hydrolysate
SEC: Size-exclusion chromatography
SGR: Specific growth rate
TH: Tuna fish hydrolysate
