# Supplementary material for: Calanus finmarchicus hydrolysate improves growth performance in feeding trial with European sea bass juveniles and increases skeletal muscle growth in cell studies
Source: Sci Rep. 2023 Jul 29;13:12295. doi: 10.1038/s41598-023-38970-5 (PMC10387114; doi:10.1038/s41598-023-38970-5)
Supplement: Supplementary file 1 — Supplementary Information. [file 41598_2023_38970_MOESM1_ESM.docx]

**APPENDIX A. Supplementary data.**

Table S1. Feed analytics, proximates, and energy.

| **As fed** | **%** | **%** | **%** | **%** | **kJ/g** |
| --- | --- | --- | --- | --- | --- |
|  | **Moisture** | **Ash** | **Protein** | **Lipid** | **Energy** |
| CALANUS | 5.38 | 6.85 | 48.28 | 16.01 | 21.69 |
| CALANUS | 5.35 | 7.35 | 48.46 | 16.16 | 21.69 |
| SARDINE | 5.50 | 6.87 | 48.12 | 15.85 | 21.82 |
| SARDINE | 5.55 | 6.85 | 48.01 | 15.81 | 21.78 |
| TUNA | 3.29 | 7.11 | 48.67 | 16.19 | 22.23 |
| TUNA | 3.33 | 7.10 | 48.70 | 16.34 | 22.19 |
| SALMON | 3.82 | 6.97 | 48.55 | 16.53 | 22.12 |
| SALMON | 3.93 | 6.84 | 48.53 | 16.42 | 22.13 |

Table S2. Feed analytics, amino acids.

| **As fed** | **%** | **%** | **%** | **%** | **%** | **%** | **%** | **%** | **%** | **%** | **%** | **%** | **%** | **%** | **%** | **%** | **%** | **%** |
| --- | --- | --- | --- | --- | --- | --- | --- | --- | --- | --- | --- | --- | --- | --- | --- | --- | --- | --- |
|  | **Arg** | **His** | **Ile** | **Leu** | **Lys** | **Thr** | **Trp** | **Val** | **Met** | **Cys** | **Phe** | **Tyr** | **Asx** | **Glx** | **Ala** | **Gly** | **Pro** | **Ser** |
| CALANUS | 2.42 | 1.03 | 1.81 | 4.18 | 2.14 | 1.64 | 0.47 | 2.15 | 0.93 | 0.61 | 2.30 | 1.68 | 3.57 | 9.84 | 2.45 | 2.07 | 3.21 | 2.18 |
| CALANUS | 2.41 | 1.05 | 1.83 | 4.15 | 2.13 | 1.66 | 0.49 | 2.17 | 1.01 | 0.60 | 2.28 | 1.71 | 3.62 | 9.81 | 2.47 | 2.09 | 3.17 | 2.19 |
| SARDINE | 2.48 | 1.00 | 1.77 | 4.13 | 2.14 | 1.66 | 0.47 | 2.06 | 1.05 | 0.61 | 2.31 | 1.71 | 3.70 | 9.21 | 2.45 | 2.14 | 3.04 | 2.22 |
| SARDINE | 2.45 | 0.98 | 1.79 | 4.10 | 2.12 | 1.68 | 0.45 | 2.07 | 1.02 | 0.59 | 2.30 | 1.68 | 3.73 | 9.29 | 2.48 | 2.12 | 3.07 | 2.20 |
| TUNA | 2.38 | 1.13 | 1.86 | 4.22 | 2.28 | 1.78 | 0.49 | 2.22 | 1.06 | 0.63 | 2.35 | 1.68 | 4.02 | 9.67 | 2.54 | 2.16 | 3.34 | 2.29 |
| TUNA | 2.39 | 1.11 | 1.85 | 4.26 | 2.30 | 1.74 | 0.48 | 2.26 | 1.05 | 0.61 | 2.36 | 1.70 | 3.95 | 9.74 | 2.51 | 2.17 | 3.33 | 2.26 |
| SALMON | 2.60 | 1.13 | 1.94 | 4.30 | 2.23 | 1.69 | 0.47 | 2.23 | 1.09 | 0.63 | 2.39 | 1.65 | 3.85 | 9.50 | 2.56 | 2.13 | 3.24 | 2.25 |
| SALMON | 2.57 | 1.12 | 1.92 | 4.31 | 2.24 | 1.70 | 0.47 | 2.22 | 1.07 | 0.62 | 2.36 | 1.67 | 3.82 | 9.56 | 2.52 | 2.12 | 3.23 | 2.23 |

Table S3. Zootechnical data from growth performance feeding trial with European sea bass, day 0-30.

|  |  |  | **g** |  | **g** |  | **%** | **g** | **g** | **g** | **g** | **%/day** | **g** |  | **%ABW/day** |  |
| --- | --- | --- | --- | --- | --- | --- | --- | --- | --- | --- | --- | --- | --- | --- | --- | --- |
| **Diet** | **Tank** | **Initial** | **Initial** | **Dead** | **Dead** | **Final** | **Survival** | **Final** | **IBW** | **FBW** | **Weight** | **SGR** | **Feed** | **FCR** | **Feed** | **PER** |
|  |  | **Fish** | **Biomass** | **Fish** | **Weight** | **Fish** |  | **Biomass** |  |  | **Gain** |  |  |  | **Intake** |  |
| CALANUS | 1 | 30 | 265 |  |  | 30 | 100 | 538 | 8.84 | 17.94 | 273 | 2.36 | 350 | 1.28 | 2.90 | 1.61 |
| CALANUS | 5 | 30 | 267 |  |  | 30 | 100 | 548 | 8.91 | 18.27 | 281 | 2.39 | 334 | 1.19 | 2.73 | 1.74 |
| CALANUS | 9 | 30 | 266 |  |  | 30 | 100 | 556 | 8.86 | 18.54 | 290 | 2.46 | 360 | 1.24 | 2.92 | 1.67 |
| SARDINE | 2 | 30 | 267 |  |  | 30 | 100 | 507 | 8.89 | 16.91 | 241 | 2.14 | 335 | 1.39 | 2.89 | 1.49 |
| SARDINE | 6 | 30 | 267 |  |  | 30 | 100 | 518 | 8.89 | 17.25 | 251 | 2.21 | 344 | 1.37 | 2.92 | 1.52 |
| SARDINE | 10 | 30 | 267 |  |  | 30 | 100 | 511 | 8.90 | 17.05 | 244 | 2.17 | 320 | 1.31 | 2.74 | 1.59 |
| TUNA | 3 | 30 | 267 |  |  | 30 | 100 | 508 | 8.90 | 16.93 | 241 | 2.14 | 316 | 1.31 | 2.72 | 1.56 |
| TUNA | 7 | 30 | 265 |  |  | 30 | 100 | 536 | 8.82 | 17.88 | 272 | 2.35 | 344 | 1.27 | 2.86 | 1.62 |
| TUNA | 11 | 30 | 266 |  |  | 30 | 100 | 519 | 8.87 | 17.29 | 253 | 2.22 | 328 | 1.30 | 2.78 | 1.58 |
| SALMON | 4 | 30 | 267 |  |  | 30 | 100 | 529 | 8.91 | 17.63 | 262 | 2.28 | 339 | 1.29 | 2.84 | 1.59 |
| SALMON | 8 | 30 | 267 |  |  | 30 | 100 | 516 | 8.90 | 17.21 | 249 | 2.20 | 334 | 1.34 | 2.84 | 1.54 |
| SALMON | 12 | 30 | 265 |  |  | 30 | 100 | 494 | 8.84 | 16.48 | 229 | 2.08 | 305 | 1.33 | 2.68 | 1.55 |

Table S4. Zootechnical data from growth performance feeding trial with European sea bass, day 0-67.

|  |  |  | **g** |  | **g** |  | **%** | **g** | **g** | **g** | **g** | **%/day** | **g** |  | **%ABW/day** |  |
| --- | --- | --- | --- | --- | --- | --- | --- | --- | --- | --- | --- | --- | --- | --- | --- | --- |
| **Diet** | **Tank** | **Initial** | **Initial** | **Dead** | **Dead** | **Final** | **Survival** | **Final** | **IBW** | **FBW** | **Weight** | **SGR** | **Feed** | **FCR** | **Feed** | **PER** |
|  |  | **Fish** | **Biomass** | **Fish** | **Weight** | **Fish** |  | **Biomass** |  |  | **Gain** |  |  |  | **Intake** |  |
| CALANUS | 1 | 30 | 265 |  |  | 30 | 100 | 973 | 8.84 | 32.44 | 708 | 1.94 | 894 | 1.26 | 2.15 | 1.64 |
| CALANUS | 5 | 30 | 267 |  |  | 30 | 100 | 950 | 8.91 | 31.68 | 683 | 1.89 | 833 | 1.22 | 2.04 | 1.70 |
| CALANUS | 9 | 30 | 266 |  |  | 30 | 100 | 991 | 8.86 | 33.04 | 725 | 1.96 | 864 | 1.19 | 2.05 | 1.73 |
| SARDINE | 2 | 30 | 267 |  |  | 30 | 100 | 908 | 8.89 | 30.25 | 641 | 1.83 | 845 | 1.32 | 2.15 | 1.58 |
| SARDINE | 6 | 30 | 267 |  |  | 30 | 100 | 913 | 8.89 | 30.45 | 647 | 1.84 | 841 | 1.30 | 2.13 | 1.60 |
| SARDINE | 10 | 30 | 267 |  |  | 30 | 100 | 895 | 8.90 | 29.82 | 627 | 1.80 | 821 | 1.31 | 2.11 | 1.59 |
| TUNA | 3 | 30 | 267 |  |  | 30 | 100 | 900 | 8.90 | 30.01 | 633 | 1.81 | 833 | 1.32 | 2.13 | 1.56 |
| TUNA | 7 | 30 | 265 |  |  | 30 | 100 | 942 | 8.82 | 31.39 | 677 | 1.89 | 845 | 1.25 | 2.09 | 1.65 |
| TUNA | 11 | 30 | 266 |  |  | 30 | 100 | 905 | 8.87 | 30.18 | 639 | 1.83 | 818 | 1.28 | 2.08 | 1.61 |
| SALMON | 4 | 30 | 267 |  |  | 30 | 100 | 958 | 8.91 | 31.93 | 691 | 1.91 | 859 | 1.24 | 2.09 | 1.66 |
| SALMON | 8 | 30 | 267 |  |  | 30 | 100 | 928 | 8.90 | 30.93 | 661 | 1.86 | 847 | 1.28 | 2.12 | 1.61 |
| SALMON | 12 | 30 | 265 | 1* | 20.40 | 29 | 97 | 884 | 8.84 | 30.48 | 630 | 1.85 | 788 | 1.25 | 2.05 | 1.65 |

*Death of fish was not associated with the diet.

Table S5. Zootechnical data from growth performance feeding trial with European sea bass, day 0-84.

|  |  |  | **g** |  | **g** |  | **%** | **g** | **g** | **g** | **g** | **%/day** | **g** |  | **%ABW/day** |  |
| --- | --- | --- | --- | --- | --- | --- | --- | --- | --- | --- | --- | --- | --- | --- | --- | --- |
| **Diet** | **Tank** | **Initial** | **Initial** | **Dead** | **Dead** | **Final** | **Survival** | **Final** | **IBW** | **FBW** | **Weight** | **SGR** | **Feed** | **FCR** | **Feed** | **PER** |
|  |  | **Fish** | **Biomass** | **Fish** | **Weight** | **Fish** |  | **Biomass** |  |  | **Gain** |  |  |  | **Intake (FI)** |  |
| CALANUS | 1 | 30 | 265 |  |  | 30 | 100 | 1169 | 8.84 | 38.96 | 904 | 1.77 | 1111 | 1.23 | 1.84 | 1.68 |
| CALANUS | 5 | 30 | 267 |  |  | 30 | 100 | 1205 | 8.91 | 40.18 | 938 | 1.79 | 1142 | 1.22 | 1.85 | 1.70 |
| CALANUS | 9 | 30 | 266 |  |  | 30 | 100 | 1179 | 8.86 | 39.29 | 913 | 1.77 | 1080 | 1.18 | 1.78 | 1.75 |
| SARDINE | 2 | 30 | 267 |  |  | 30 | 100 | 1087 | 8.89 | 36.23 | 820 | 1.67 | 1063 | 1.30 | 1.87 | 1.60 |
| SARDINE | 6 | 30 | 267 |  |  | 30 | 100 | 1003 | 8.89 | 33.44 | 736 | 1.58 | 981 | 1.33 | 1.84 | 1.56 |
| SARDINE | 10 | 30 | 267 |  |  | 30 | 100 | 1044 | 8.90 | 34.79 | 777 | 1.62 | 1053 | 1.36 | 1.91 | 1.53 |
| TUNA | 3 | 30 | 267 |  |  | 30 | 100 | 1090 | 8.90 | 36.32 | 822 | 1.67 | 1083 | 1.32 | 1.90 | 1.56 |
| TUNA | 7 | 30 | 265 |  |  | 30 | 100 | 1127 | 8.82 | 37.57 | 862 | 1.72 | 1094 | 1.27 | 1.87 | 1.62 |
| TUNA | 11 | 30 | 266 |  |  | 30 | 100 | 1090 | 8.87 | 36.34 | 824 | 1.68 | 1047 | 1.27 | 1.84 | 1.62 |
| SALMON | 4 | 30 | 267 |  |  | 30 | 100 | 1149 | 8.91 | 38.29 | 881 | 1.74 | 1131 | 1.28 | 1.90 | 1.61 |
| SALMON | 8 | 30 | 267 |  |  | 30 | 100 | 1092 | 8.90 | 36.38 | 825 | 1.68 | 1045 | 1.27 | 1.83 | 1.63 |
| SALMON | 12 | 30 | 265 | 1* | 20.40 | 29 | 97 | 1120 | 8.84 | 38.63 | 867 | 1.76 | 1094 | 1.26 | 1.88 | 1.63 |

*Death of fish was not associated with the diet.

Table S6. Whole-body composition of fish at end of trial (84 days).

|  |  | **CALANUS** |  | **SARDINE** |  | **TUNA** |  | **SALMON** |  |
| --- | --- | --- | --- | --- | --- | --- | --- | --- | --- |
| Moisture, % |  | 65.8 ± 0.8 |  | 65.5 ± 1.2 |  | 65.6 ± 1.4 |  | 64.9 ± 0.4 |  |
| Protein, % |  | 17.5 ± 0.5 |  | 18.2 ± 0.4 |  | 18.0 ± 0.1 |  | 17.8 ± 0.4 |  |
| Fat, % |  | 12.2 ± 0.8 |  | 12.0 ± 0.7 |  | 12.2 ± 1.3 |  | 12.6 ± 0.4 |  |
| Ash, % |  | 4.0 ± 0.3 |  | 3.8 ± 0.2 |  | 3.9 ± 0.2 |  | 3.9 ± 0.3 |  |
| Energy, kJ/g |  | 8.4 ± 0.4 |  | 8.3 ± 0.3 |  | 8.4 ± 0.5 |  | 8.6 ± 0.2 |  |

Values are means ± standard deviation (n=3).

Absence of superscripts within a row, denotes the absence of statistical differences (P>0.05).

Table S7.1. Summary of one-way ANOVA Day 30.

| **Table Analyzed** | **Body weight 30** | **SGR 30** | **FCR 30** | **PER 30** |
| --- | --- | --- | --- | --- |
|  |  |  |  |  |
| **ANOVA summary** |  |  |  |  |
| F | 5,285 | 5,323 | 6,249 | 5,203 |
| P value | 0,0266 | 0,0261 | 0,0172 | 0,0277 |
| P value summary | * | * | * | * |
| Significant diff. among means (P < 0.05)? | Yes | Yes | Yes | Yes |
| R squared | 0,6646 | 0,6662 | 0,7009 | 0,6611 |
|  |  |  |  |  |
| **Brown-Forsythe test** |  |  |  |  |
| F (DFn, DFd) | 0,6567 (3, 8) | 0,6827 (3, 8) | 0,3285 (3, 8) | 0,5206 (3, 8) |
| P value | 0,6012 | 0,5871 | 0,8051 | 0,6799 |
| P value summary | ns | ns | ns | ns |
| Are SDs significantly different (P < 0.05)? | No | No | No | No |
|  |  |  |  |  |
| **Data summary** |  |  |  |  |
| Number of treatments (columns) | 4 | 4 | 4 | 4 |
| Number of values (total) | 12 | 12 | 12 | 12 |

| **ANOVA table Body weight 30** | **SS** | **DF** | **MS** | **F (DFn, DFd)** | **P value** |
| --- | --- | --- | --- | --- | --- |
| Treatment (between columns) | 2,728 | 3 | 0,9092 | F (3, 8) = 5,285 | P=0,0266 |
| Residual (within columns) | 1,376 | 8 | 0,1720 |  |  |
| Total | 4,104 | 11 |  |  |  |
|  |  |  |  |  |  |
| **ANOVA table SGR 30** | **SS** | **DF** | **MS** | **F (DFn, DFd)** | **P value** |
| Treatment (between columns) | 0,1007 | 3 | 0,03358 | F (3, 8) = 5,323 | P=0,0261 |
| Residual (within columns) | 0,05047 | 8 | 0,006308 |  |  |
| Total | 0,1512 | 11 |  |  |  |
|  |  |  |  |  |  |
| **ANOVA table FCR 30** | **SS** | **DF** | **MS** | **F (DFn, DFd)** | **P value** |
| Treatment (between columns) | 0,02297 | 3 | 0,007656 | F (3, 8) = 6,249 | P=0,0172 |
| Residual (within columns) | 0,009800 | 8 | 0,001225 |  |  |
| Total | 0,03277 | 11 |  |  |  |
|  |  |  |  |  |  |
| **ANOVA table PER 30** | **SS** | **DF** | **MS** | **F (DFn, DFd)** | **P value** |
| Treatment (between columns) | 0,03317 | 3 | 0,01106 | F (3, 8) = 5,203 | P=0,0277 |
| Residual (within columns) | 0,01700 | 8 | 0,002125 |  |  |
| Total | 0,05017 | 11 |  |  |  |

Table S7.2. Summary of one-way ANOVA Day 67.

| **Table Analyzed** | **Body weight 67** | **SGR 67** | **FCR 67** | **PER 67** |
| --- | --- | --- | --- | --- |
|  |  |  |  |  |
| **ANOVA summary** |  |  |  |  |
| F | 6,725 | 5,756 | 5,496 | 4,730 |
| P value | 0,0141 | 0,0214 | 0,0241 | 0,0350 |
| P value summary | * | * | * | * |
| Significant diff. among means (P < 0.05)? | Yes | Yes | Yes | Yes |
| R squared | 0,7160 | 0,6834 | 0,6733 | 0,6395 |
|  |  |  |  |  |
| **Brown-Forsythe test** |  |  |  |  |
| F (DFn, DFd) | 0,2232 (3, 8) | 0,1556 (3, 8) | 0,7059 (3, 8) | 0,7473 (3, 8) |
| P value | 0,8777 | 0,9232 | 0,5749 | 0,5537 |
| P value summary | ns | ns | ns | ns |
| Are SDs significantly different (P < 0.05)? | No | No | No | No |
|  |  |  |  |  |
| **Data summary** |  |  |  |  |
| Number of treatments (columns) | 4 | 4 | 4 | 4 |
| Number of values (total) | 12 | 12 | 12 | 12 |

| **ANOVA table Body weight 67** | **SS** | **DF** | **MS** | **F (DFn, DFd)** | **P value** |
| --- | --- | --- | --- | --- | --- |
| Treatment (between columns) | 8,499 | 3 | 2,833 | F (3, 8) = 6,725 | P=0,0141 |
| Residual (within columns) | 3,370 | 8 | 0,4213 |  |  |
| Total | 11,87 | 11 |  |  |  |
|  |  |  |  |  |  |
| **ANOVA table SGR 67** | **SS** | **DF** | **MS** | **F (DFn, DFd)** | **P value** |
| Treatment (between columns) | 0,01942 | 3 | 0,006475 | F (3, 8) = 5,756 | P=0,0214 |
| Residual (within columns) | 0,009000 | 8 | 0,001125 |  |  |
| Total | 0,02842 | 11 |  |  |  |
|  |  |  |  |  |  |
| **ANOVA table FCR 67** | **SS** | **DF** | **MS** | **F (DFn, DFd)** | **P value** |
| Treatment (between columns) | 0,01237 | 3 | 0,004122 | F (3, 8) = 5,496 | P=0,0241 |
| Residual (within columns) | 0,006000 | 8 | 0,0007500 |  |  |
| Total | 0,01837 | 11 |  |  |  |
|  |  |  |  |  |  |
| **ANOVA table PER 67** | **SS** | **DF** | **MS** | **F (DFn, DFd)** | **P value** |
| Treatment (between columns) | 0,01750 | 3 | 0,005833 | F (3, 8) = 4,730 | P=0,0350 |
| Residual (within columns) | 0,009867 | 8 | 0,001233 |  |  |
| Total | 0,02737 | 11 |  |  |  |

Table S7.3. Summary of one-way ANOVA Day 84.

| **Table Analyzed** | **Body weight 84** | **SGR 84** | **FCR 84** | **PER 84** |
| --- | --- | --- | --- | --- |
|  |  |  |  |  |
| **ANOVA summary** |  |  |  |  |
| F | 10,63 | 10,87 | 11,70 | 12,05 |
| P value | 0,0036 | 0,0034 | 0,0027 | 0,0024 |
| P value summary | ** | ** | ** | ** |
| Significant diff. among means (P < 0.05)? | Yes | Yes | Yes | Yes |
| R squared | 0,7995 | 0,8030 | 0,8144 | 0,8189 |
|  |  |  |  |  |
| **Brown-Forsythe test** |  |  |  |  |
| F (DFn, DFd) | 0,3385 (3, 8) | 0,6061 (3, 8) | 0,2500 (3, 8) | 0,3148 (3, 8) |
| P value | 0,7983 | 0,6294 | 0,8592 | 0,8145 |
| P value summary | ns | ns | ns | ns |
| Are SDs significantly different (P < 0.05)? | No | No | No | No |
|  |  |  |  |  |
| **Data summary** |  |  |  |  |
| Number of treatments (columns) | 4 | 4 | 4 | 4 |
| Number of values (total) | 12 | 12 | 12 | 12 |

| **ANOVA table Body weight 84** | **SS** | **DF** | **MS** | **F (DFn, DFd)** | **P value** |
| --- | --- | --- | --- | --- | --- |
| Treatment (between columns) | 34,45 | 3 | 11,48 | F (3, 8) = 10,63 | P=0,0036 |
| Residual (within columns) | 8,641 | 8 | 1,080 |  |  |
| Total | 43,09 | 11 |  |  |  |
|  |  |  |  |  |  |
| **ANOVA table SGR 84** | **SS** | **DF** | **MS** | **F (DFn, DFd)** | **P value** |
| Treatment (between columns) | 0,03749 | 3 | 0,01250 | F (3, 8) = 10,87 | P=0,0034 |
| Residual (within columns) | 0,009200 | 8 | 0,001150 |  |  |
| Total | 0,04669 | 11 |  |  |  |
|  |  |  |  |  |  |
| **ANOVA table FCR 84** | **SS** | **DF** | **MS** | **F (DFn, DFd)** | **P value** |
| Treatment (between columns) | 0,02223 | 3 | 0,007408 | F (3, 8) = 11,70 | P=0,0027 |
| Residual (within columns) | 0,005067 | 8 | 0,0006333 |  |  |
| Total | 0,02729 | 11 |  |  |  |
|  |  |  |  |  |  |
| **ANOVA table PER 84** | **SS** | **DF** | **MS** | **F (DFn, DFd)** | **P value** |
| Treatment (between columns) | 0,03496 | 3 | 0,01165 | F (3, 8) = 12,05 | P=0,0024 |
| Residual (within columns) | 0,007733 | 8 | 0,0009667 |  |  |
| Total | 0,04269 | 11 |  |  |  |

Table S8. Primers for gene expression analysis.

| **Gene** | **5’-3’** | **Genbank accession no.** |
| --- | --- | --- |
| *ef1a* | CACCACCGGCCATCTGATCTACAA | AF321836 |
|  | TCAGCAGCCTCCTTCTCGAACTTC |  |
| *rpol2* | TAACGCCTGCCTCTTCACGTTGA | CA049789 |
|  | ATGAGGGACCTTGTAGCCAGCAA |  |
| *eif3* | CAGGATGTTGTTGCTGGATGGG | DW542195 |
|  | ACCCAACTGGGCAGGTCAAGA |  |
| *myog* | ATTGAGAGGCTGCAGGCACTTG | DQ294029 |
|  | GTGCGGTAGTGTAAGCCCTGTGTT |  |
| *mrf4* | CCTTTGTACCACGGGAATGACAGC | DQ479952 |
|  | TGTCGGTCGGTGCAGACTTTCTT |  |
| *myf5* | GGGAACTGGATGGCTCAGA | DQ452070 |
|  | TGCTGGACTTACGCTTGCA |  |
| *myod1a* | GCTATATTGAGTCTCTCCAAGGCCTGC | AJ557148 |
|  | GGACAAGACGGGTCATTGAAATCC |  |
| *mhc* | AGAAGCACGCCACTGAAAAC | DN164736 |
|  | AACCCTCAAGGTCGTCCACT |  |
| *mlc2* | CCATCAACTTCACCGTCTTCCTCAC | NM_001123716 |
|  | CAGCCCACAGGTTCTTCATCTCC |  |
| *igf2b* | TGTGCCAAACCTGCCAAGT | AY049955 |
|  | CCTTCCTCTGCCACACCTCATAT |  |
| *pax7* | AAGGTCGGGTGAACCAGCT | AJ618975 |
|  | GAGATTACGCAGGGTCGGAT |  |
| *pcna* | GGGAGGAGGATCTTGTTGGC | BT045597 |
|  | ACGCAATAGCGATGCCATGA |  |
| *sdc4* | TCATGAGCTCTATCGGCAGC | XM_014165901.1 |
|  | ACGCATCCAGATAGTTGGGC |  |


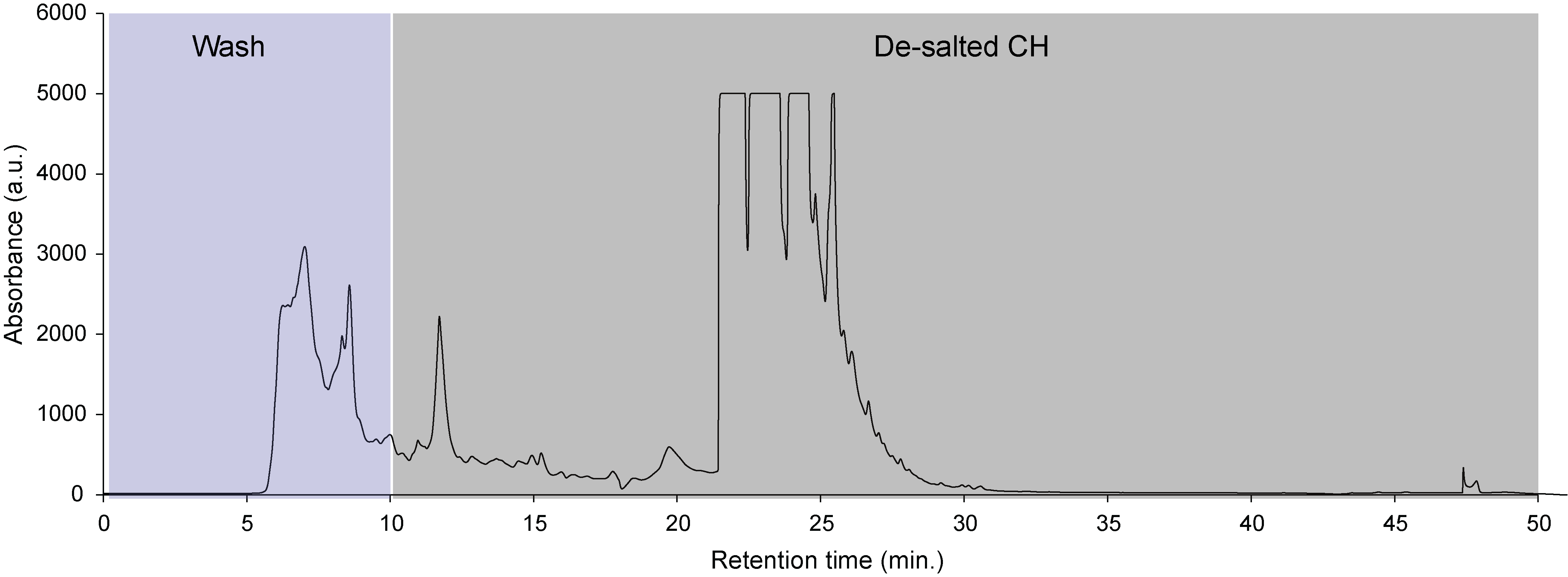


Figure S1. Semi-preparative reversed phase chromatogram of crude CH (214 nm). Chromatographic regions were highlighted to indicate how desalted CH was acquired.


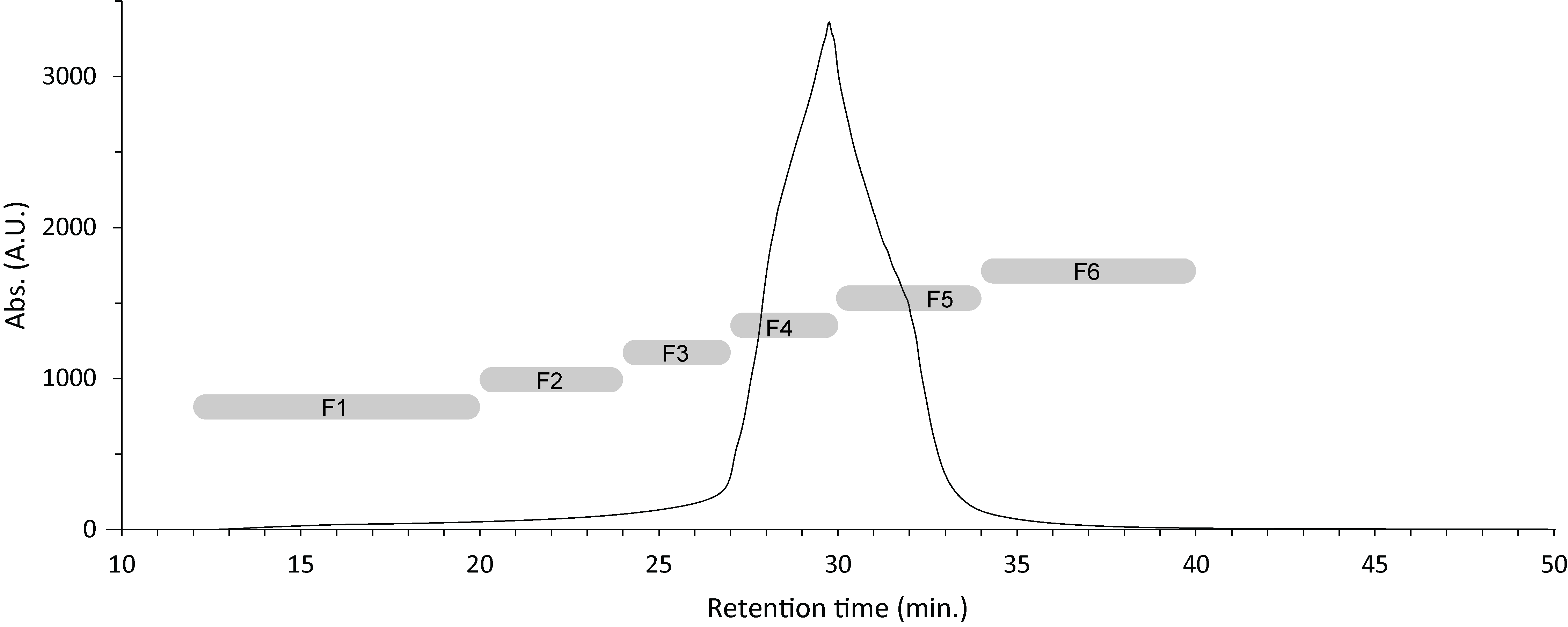


Figure S2. Semi-preparative size exclusion chromatogram of crude CH (214 nm). Chromatographic regions were F1-F6 were collected is highlighted in gray.
